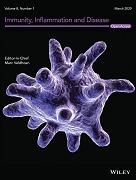# Issue Information

**DOI:** 10.1002/iid3.256

**Published:** 2020-02-13

## Abstract